# Aerosolizable Lipid Nanoparticles for Pulmonary Delivery of mRNA through Design of Experiments

**DOI:** 10.3390/pharmaceutics12111042

**Published:** 2020-10-30

**Authors:** Hairui Zhang, Jasmim Leal, Melissa R. Soto, Hugh D. C. Smyth, Debadyuti Ghosh

**Affiliations:** Division of Molecular Pharmaceutics and Drug Delivery, College of Pharmacy, The University of Texas at Austin, Austin, TX 78712, USA; hrzhang@utexas.edu (H.Z.); jasmim.leal@utexas.edu (J.L.); melissasoto93@utexas.edu (M.R.S.); hugh.smyth@austin.utexas.edu (H.D.C.S.)

**Keywords:** gene therapy, mRNA therapeutics, lipid nanoparticles, pulmonary delivery, aerosolization

## Abstract

Messenger RNA is a class of promising nucleic acid therapeutics to treat a variety of diseases, including genetic diseases. The development of a stable and efficacious mRNA pulmonary delivery system would enable high therapeutic concentrations locally in the lungs to improve efficacy and limit potential toxicities. In this study, we employed a Design of Experiments (DOE) strategy to screen a library of lipid nanoparticle compositions to identify formulations possessing high potency both before and after aerosolization. Lipid nanoparticles (LNPs) showed stable physicochemical properties for at least 14 days of storage at 4 °C, and most formulations exhibited high encapsulation efficiencies greater than 80%. Generally, upon nebulization, LNP formulations showed increased particle size and decreased encapsulation efficiencies. An increasing molar ratio of poly-(ethylene) glycol (PEG)-lipid significantly decreased size but also intracellular protein expression of mRNA. We identified four formulations possessing higher intracellular protein expression ability in vitro even after aerosolization which were then assessed in in vivo studies. It was found that luciferase protein was predominately expressed in the mouse lung for the four lead formulations before and after nebulization. This study demonstrated that LNPs hold promise to be applied for aerosolization-mediated pulmonary mRNA delivery.

## 1. Introduction

Successful delivery of nucleic acids for gene replacement therapy and editing offers the promise of long-term correction and a cure for genetic diseases affecting the lung, such as cystic fibrosis (CF) and alpha-1 antitrypsin deficiency. However, it is a challenge to systemically deliver gene therapies at sufficient therapeutic concentrations at the site of action. Pulmonary delivery of therapeutics is attractive due to local deposition at high concentrations of medicines directly within the lungs [1]. Polymer and lipid non-viral delivery platforms [2,3] are promising carriers to protect and encapsulate these larger sized nucleic acid payloads with less immunogenicity compared to viral vectors such as adeno-associated virus [4,5]. In particular, lipid nanoparticles (LNPs) for delivery of nucleic acids such as messenger RNA (mRNA) are promising gene therapies, and recent studies have demonstrated that LNPs could be used for pulmonary delivery of mRNA [6,7]. Cheng et al. showed that adding varying concentrations of permanently cationic or anionic lipids to conventional LNPs resulted in nanoparticles with selective organ targeting (SORT) [6]. Specifically, they observed that intravenously administered LNPs with a molar percentage of 50% 1,2-dioleoyl-3-trimethylammonium-propane (DOTAP) achieved selective targeting to the lung. In another study, Kim et al. investigated in vivo transfection of LNPs to the nasal respiratory epithelia [7]. After intranasal instillation of LNPs encapsulating luciferase reporter mRNA, they primarily observed luciferase expression around the nasal cavity of tested mice, with minor expression in the lungs. Lastly, Translate Bio, a company focusing on mRNA therapeutics, developed LNPs that carry cystic fibrosis transmembrane conductance regulator (CFTR) mRNA (MRT5005), and is the first company to execute a clinical trial investigating nebulized delivery of mRNA treatment [8]. While these studies reflect the ability of LNPs to be delivered to the lungs, there remain no reports using Design of Experiments (DOE) methodologies to investigate the stability and delivery post nebulization of different compositions of LNP libraries in order to achieve efficient mRNA expression in the lungs.

Pulmonary delivery of mRNA is a promising nucleic acid therapeutic approach to replace dysfunctional proteins or modify gene sequences by delivering gene-editing components locally to the lung [9,10,11,12]. Compared to DNA-based therapeutics, mRNA therapeutics can achieve transient protein expression without requiring entry into the target cell nucleus and can circumvent any off-target effects due to possible integration into the host genome. However, the delivery of naked mRNA is not therapeutically viable, as it is prone to rapid degradation by endogenous nucleases and may also induce immune responses. Moreover, nucleic acids possess an overall negative charge, which limits their ability to cross cellular membranes for delivery. Therefore, a delivery system for mRNA tends to have narrow design window criteria. The delivery system should provide stability and low immunogenicity, while retaining the potency of mRNA for efficient intracellular delivery [13,14].

LNPs specifically can address the aforementioned challenges of RNA-mediated delivery. LNPs commonly consist of ionizable lipids, phospholipids, cholesterol, and poly-(ethylene) glycol (PEG)-lipid. The cationic, ionizable lipids help condense nucleic acids to form LNPs and have been successfully translated toward clinical therapies [15]. Recently, the approval of the first small interfering RNA drug for the treatment of hereditary transthyretin amyloidosis, ONPATTRO^®^ (patisiran), illustrates the clinical feasibility and application of LNP-based gene delivery [16,17]. Although the LNP-based delivery of short RNA (e.g., siRNA) has been investigated for many years, the feasibility of LNP-mediated mRNA delivery has only been explored recently [18,19,20,21,22,23,24].

For pulmonary gene delivery, LNPs offer numerous advantages. Using biodegradable lipids, LNPs can be tolerated by the airways without incurring toxicity [25]. The size of LNPs can be controlled into the nanometer range, which allows them to be entrapped in aerosolized droplets that can easily be deposited deep in the lungs [26]. In addition, the small size of LNPs may enable a longer residency time in the airways due to adherence to the mucosal surface [26,27]. However, to achieve clinical translation of LNP-mRNA for pulmonary delivery, LNP-mRNA formulations must be stably aerosolized. The potency of LNP formulations is heavily influenced by particular LNP compositions [28], and it is a challenge to screen, test, and identify numerous LNP-mRNA formulations with the desired compositions that would allow for stability after nebulization. Recently, a DOE approach has been used to screen LNP formulations to optimize LNP-based mRNA delivery, which reduces the number of individual experiments needed for the screening process [29]. Here, we used a DOE strategy to optimize LNP formulations for mRNA delivery to the lungs. To explore the feasibility of aerosolized LNP-mRNA and the effects of LNP compositions on the potency of aerosolized LNP formulations, we employed a custom mixture design with constraints to determine the influence of the different components on physicochemical properties such as size, zeta-potential, pKa, encapsulation efficiencies, as well as in vitro intracellular protein expression and in vivo pulmonary delivery.

## 2. Materials and Methods

### 2.1. Materials

(6Z,9Z,28Z,31Z)-heptatriacont-6,9,28,31-tetraene-19-yl 4-(dimethylamino)butanoate (DLin-MC3-DMA) was purchased from Biofine International Inc., (Vancouver, BC, Canada). 1,2-dipalmitoyl-sn-glycero-3-phosphocholine (DPPC), 1,2-dimyristoyl-rac-glycero-3-methoxypolyethylene glycol-2000 (DMG-PEG-2000), 1,2-distearoyl-sn-glycero-3- phosphocholine (DSPC), 1,2-distearoyl-sn-glycero-3-phosphoethanolamine-N-[Amino (Polyethylene Glycol) 2000 (DSPE-PEG 2000), and (Delta 9 cis)/1,2-dioleoyl-sn-glycero-3-phosphoethanolamine (DOPE) were purchased from Avanti Polar Lipids, AL, USA. N-(methylpolyoxyethyleneoxycarbonyl)-1,2-dimyristoyl-sn-glycero-3-phosphoehtanolamine (DMPE-PEG 2000) was purchased from NOF Corporation, Tokyo, Japan. Cholesterol was purchased from Sigma Aldrich, (St. Louis, MO, USA). Ethanol (molecular grade) was purchased from Decon Laboratories, Inc., (King of Prussia, PA, USA). CleanCap^®^ Enhanced Green Fluorescent Protein (EGFP) mRNA and CleanCap^®^ Firefly luciferase (FLuc) mRNA were purchased from TriLink, San Diego, CA, USA. HEK-293 (CRL-1573) and NuLi-1 cells (CRL-4011) were purchased from American Type Culture Collection (ATCC) (Manassas, VA, USA). Slide-A-LyzerTM Gamma Irradiated Dialysis Cassette (10kDa), Quanit-iT™ RiboGreen^®^ RNA Reagent and Kit (Invitrogen), and Opti-MEM™ Reduced Serum Media (Gibco) were purchased from ThermoFisher Scientific Inc., Waltham, MA, USA. Dulbecco’s Modification of Eagle’s Medium (DMEM), Fetal Bovine Serum (FBS), and Penicillin/Streptomycin (100X) were purchased from Corning, Manassas, VA, USA. Balb/c mice were purchased from Charles River Laboratories, Inc, Wilmington, MA, USA.

### 2.2. Methods

#### 2.2.1. Preparation of LNP Formulations

Lipid nanoparticles containing EGFP mRNA or FLuc mRNA were prepared by combining an aqueous phase (mRNA diluted in 100 mM sodium citrate buffer, pH 3.0) and an organic phase containing ethanol and lipids according to each formulation (Table S2) using a microfluidic mixer (Precision Nanosystems, Vancouver, Canada) [30]. After preparation, LNP formulations were dialyzed into 1X phosphate buffered saline (PBS) (pH 7.4) for 2 h in 10K MWCO Slide-A-Lyzer dialysis cassettes (Thermo Fisher Scientific, Waltham, MA, USA).

#### 2.2.2. Measurements of Size and Zeta Potential

The size and zeta potential of LNP formulations were characterized by using Zetasizer Nano-ZS (Malvern Instruments MA, USA). Each formulation was 10-fold diluted in 0.1X PBS buffer (pH 7.4) for size measurement and 40-fold diluted in 0.1X PBS (pH 7.4) for zeta potential measurement. Dynamic light scattering was performed on diluted samples at 25 °C with 173° and the reported z-average diameter is a mean of three measurements.

#### 2.2.3. mRNA Encapsulation Efficiency

mRNA encapsulation efficiency was evaluated by low range Quanti-iT RiboGreen RNA reagent assay (Thermo Fisher Scientific, Waltham, MA, USA). Each LNP sample was diluted into Tris-EDTA (TE) buffer down to a mRNA concentration of 0.2 ng/µL. Aliquots of each LNP working solution were further diluted 1:1 in TE buffer (measuring unencapsulated mRNA) or 1:1 in TE buffer with 4% Triton-X100 (measuring total mRNA-both encapsulated within LNPs and unencapsulated free mRNA) in a 96-well plate. Samples were prepared in duplicate and 100 µL of 2000-fold diluted Quanti-iT™ RiboGreen RNA reagent was added to each sample the fluorescence intensity was measured by plate reader at excitation and emission wavelengths of 480 and 520 nm (Infinite M200, Tecan, Männedorf, Switzerland), respectively.

#### 2.2.4. 6-p-Toluidino-2-Naphthalenesulfonic Acid (TNS) Assays

The pKa of the LNP formulations was determined as previously described [28]. Briefly, a series of buffers with pH ranging from 2.5 to 11 (pH 2.5, pH 3, pH 3.5, pH 4, pH 4.6, pH 5, pH 5.5, pH 5.8, pH 6, pH 6.5, pH 7, pH 7.5, pH 8, pH 8.5, pH 9, pH 9.5, pH 10, pH 10.5, pH 11) were prepared by adjusting the pH of each buffer solution consisting of 10 mM (4-(2-hydroxyethyl)-1-piperazineethanesulfonic acid) HEPES, 10 mM 2-(4-Morpholino)ethanesulfonic acid MES, 10 mM ammonium acetate, 130 mM sodium chloride (NaCl) with 1 N hydrochloric acid (HCl). Additionally, 90 μL of each buffer solution was added to a 96-well plate. After, 2 μL of TNS stock solution (300 μM in dimethyl sulfoxide (DMSO)) was added to the buffer solutions in the 96-well plate. Then, 3 μL of each LNP formulation (prepared with mRNA) were added to the above mixture. The fluorescence intensity was measured at an excitation wavelength of 325 nm and an emission wavelength of 435 nm. The fluorescence intensity was plotted against pH values and fitted using a three-parameter logistic equation (GraphPad Prism v.6, GraphPad Software, San Diego, CA, USA). The pH value at which half of the maximum fluorescence is reached was calculated as the pKa of LNP formulations.

#### 2.2.5. Aerosolization of LNP Formulations

It has been shown that vibrating mesh nebulizers can be used to aerosolize shear susceptible formulations such as liposomes and niosomes and therefore are a good alternative to air jet and ultrasound nebulizers [31,32]. Approximately 500 µL of LNPs were loaded onto the reservoir of the Aerogen Solo device (Aerogen Ltd., Galway Ireland), which is a vibrating mesh nebulizer. Next, the nebulization cycle was started on the Aerogen Pro-X Controller (Aerogen Ltd., Galway Ireland), and a 1.5 mL microcentrifuge tube (pre-chilled on ice) was placed close to the vibrating mesh outlet to collect the nebulized LNPs. Nebulization was stopped once we observed that no more aerosol was being produced. The nebulizer was cleaned in between formulations by nebulizing 1 mL of ultrapure water.

#### 2.2.6. Cell Culture

Human embryonic kidney 293 (HEK-293) cells were cultured with Dulbecco’s Modified Eagle Medium containing 10% FBS and 1% penicillin streptomycin. NuLi-1 cells (ATCC CRL-4013) were cultured in flasks pre-coated with 60 μg/mL solution of human placental collagen type IV (Sigma Aldrich, St. Louis, MO, USA) and grown in bronchial epithelial growth medium (BEGM) supplemented with SingleQuot additives from Lonza (BEGM Bullet Kit, reference CC-3170) and 50 μg/mL G-418. All cell lines were maintained as monolayer cultures at 37 °C and 5% CO_2_.

#### 2.2.7. Intracellular Protein Expression In Vitro

Cells were seeded in 96-well plates at a cell density of 12,500 cells/well and grown for 24 h at 37 °C and 5% CO_2_. Then 10 µL of LNP at a 10 ng EGFP mRNA/µL concentration was added to cells in 0.2 mL cell culture media for 24 h. After, the cell culture media was removed, and cells were washed with 1X PBS. To detach the cells, 100 µL of 0.25% trypsin-EDTA solution was added to each well and incubated at 37 °C for 8–10 min. Next, 100 µL of 1% FBS in Dulbecco’s phosphate buffered saline were added, cells were spun down at 125× *g* for 5 to 10 min and the supernatant was discarded. Cells were resuspended in 50 µL 1X PBS with 0.25 µL propidium iodide (PI) (1 mg/mL) solution. Cell percent GFP expression (i.e., transfection efficiency) and fluorescence intensity were analyzed by flow cytometry (5000 events were collected). To calculate % GFP expression, a negative control (non-GFP expressing cells) was used to gate the GFP positive population using FlowJo software (BD Life Sciences, Franklin Lakes, NJ, USA). NuLi-1 cells (catalog number CRL-4011) were purchased from ATCC (Manassas, VA, USA).

#### 2.2.8. In Vivo Transfection

All animal protocols were approved by the Institutional Animal Care and Use Committee (IACUC) at the University of Texas at Austin under IACUC protocol number AUP-2017-00070, approval date 21 April 2017. Balb/c mice (female, 6-8 weeks) were anesthetized under a continuous flow of 2% isoflurane, and approximately 50 µL of LNP containing 1.5 µg of FLuc mRNA in PBS were administered intratracheally. After 6 h, mice were intraperitoneally (i.p.) injected with D-Luciferin solution (30 mg/mL) to reach 150 mg Luciferin/kg body weight. After 15 min, mice were sacrificed and the lungs were carefully harvested and imaged by an In Vivo Imaging System (IVIS), with bioluminescence setting and a luminescent exposure time of 60 s. Quantification of luminescence (in radiance [p/sec/cm^2^/sr]) was performed with Living Image 4.3 software (PerkinElmer, Waltham, MA, USA).

#### 2.2.9. Statistical Analysis

Design of Experiments (DOE) and the statistical analysis were performed using JMP 13 statistical software (JMP, SAS Institute, Cary, NC, USA). Data values are expressed as mean ± standard deviations (SD). When required, one-way analysis of variance (one-way ANOVA) or Student’s *t*-test was performed. * *p*-values < 0.05, ** *p*-values < 0.01, *** *p*-values < 0.001, and **** *p*-values < 0.0001 were considered statistically significant.

## 3. Results

### 3.1. Effects of N/P Molar Ratio on the Efficacy of LNP Formulations and Mixture Experimental Design with Constraints

To establish a baseline and initially identify the optimal N/P (amine group to phosphate group) molar ratio desired for intracellular protein expression, six LNP formulations encapsulating EGFP mRNA were prepared by varying the N/P ratio between 6:1 to 200:1, using as reference a commercial formulation as previously published [28,33] for siRNA delivery in vivo. LNP formulations were composed of DLin-MC3-DMA, a phosphatidylcholine (1,2-distearoyl-sn-glycero-3-phosphocholine, DSPC), cholesterol, and a PEG-lipid (polyethylene glycol-dimyristolglycerol, PEG-DMG) at a single molar ratio of 50:10:38.5:1.5, respectively. The different N/P ratios (N/P = 6:1, 15:1, 30:1, 50:1, 100:1, and 200:1) were achieved by varying the relative amount of lipid composition added to the mRNA (10 ng/µL). LNPs were prepared and the intracellular protein expression of each formulation was evaluated in HEK-293 cells by flow cytometry. As shown in Figure S1, the LNP formulation with an N/P ratio = 15:1 demonstrated both the highest percent GFP expression and mean fluorescence intensity. The intracellular protein expression decreased as the N/P ratio increased from 15:1 to 200:1. The N/P ratio = 15:1 was maintained for the following experiments, and this particular formulation is subsequently used in the experiments as the “reference formulation”.

LNPs consist generally of four lipid components: ionizable lipid, phospholipid, PEG-lipid, and cholesterol. The different types and amount of lipids may affect the transfection efficacy of LNP formulations. In order to systematically investigate the effects of the variables on the potency of LNP formulations, a mixture design with constraints was employed in this study (Table S1). Using JMP statistical software, a design of 18 LNP formulations was generated for testing (Table S2).

### 3.2. Characterization of mRNA-LNPs

Based on a mixture design with constraints, 18 formulations with an N/P ratio = 15:1 were prepared using the NanoAssemblr^®^ benchtop system. The size and zeta potential of the LNPs were evaluated by Zetasizer Nano. Size and zeta potential measurements were performed in 0.1× PBS at 25 °C and a scattering angle of 173°. As shown in Figure S2a, the particle size of the LNP formulations before nebulization varied from 35.7 ± 1.1 nm (F14) to 120.9 ± 3.4 nm (F8), while the zeta potential ranged from −12.2 ± 5.5 mV (F3) to 18.8 ± 1.2 mV (F13) (Figure S2b). Furthermore, the size and zeta potential of the LNP formulations did not show significant changes after 14 days of storage in 4 °C, which indicated that the size and surface charge of all formulations remained stable for at least 2 weeks (Figure S2a,b). The polydispersity index (PDI) of the LNPs ranged from 0.035 ± 0.029 to 0.261 ± 0.005 at Day 1 and the PDI of LNPs raged from 0.082 ± 0.010 to 0.486 ± 0.029 at Day 14 (Table S3). The encapsulation efficiency of the formulations was evaluated by RiboGreen assay according to the manufacturer protocol (Thermo Fisher Scientific, Waltham, MA, USA). Most of the formulations possessed a high encapsulation efficiency greater than 80%, except for F12 which showed 49% encapsulation efficiency (Figure S2c). It has been previously reported that the pKa of LNPs may be critical for endosomal escape and has been implicated as a correlator for in vivo efficacy of gene therapy [28]. Therefore, we measured the pKa of LNP formulations loaded with EGFP mRNA using the TNS assay and found that the LNP pKa values ranged from 5.74 (F15) to 6.11 (F14) (Figure S2d).

**Figure 1:**
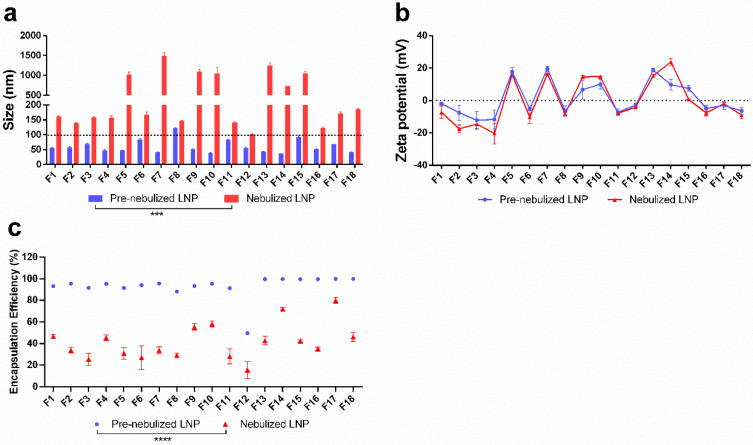
Stability of LNP formulations before and after nebulization in terms of (**a**) size, (**b**) zeta-potential, and (**c**) encapsulation efficiency. The size (*** *p* = 0.0004) and encapsulation efficiency (**** *p* < 0.0001) of nebulized formulation were significantly different from pre-nebulized formulations.

To translate LNP formulations for pulmonary delivery, they must be aerosolized without significant instability. Towards that end, we investigated the effects of nebulization on the LNP formulations and subsequently identified the formulations that retained high intracellular protein expression in vitro following nebulization. LNP formulations were aerosolized by the Aerogen Solo (Aerogen Ltd., Galway, Ireland) nebulizer and the potency of each nebulized formulation was evaluated in HEK-293 and human bronchial epithelial NuLi-1 cell lines. After nebulization, the size of the LNP formulations ranged from 100.9 nm (F12) to 1480.7 nm (F7) and showed a significant increase compared to the pre-nebulized LNP formulations (Figure 1a), while the zeta potential showed no significant changes amongst all formulations (Figure 1b). It is worth noting that F8 had the smallest change in size upon nebulization, and F7 showed the largest change in size after nebulization. The encapsulation efficiency of the LNP formulations significantly decreased after nebulization as shown in Figure 1c, which indicated that the mRNA potentially leaked from the LNPs upon the nebulization process. The encapsulation efficiency of nebulized LNP formulations ranged from 15.5% (F12) to 79.9% (F17).

### 3.3. Intracellular Protein Expression of LNP Formulations in HEK-293 and NuLi-1 Cells

**Figure 2:**
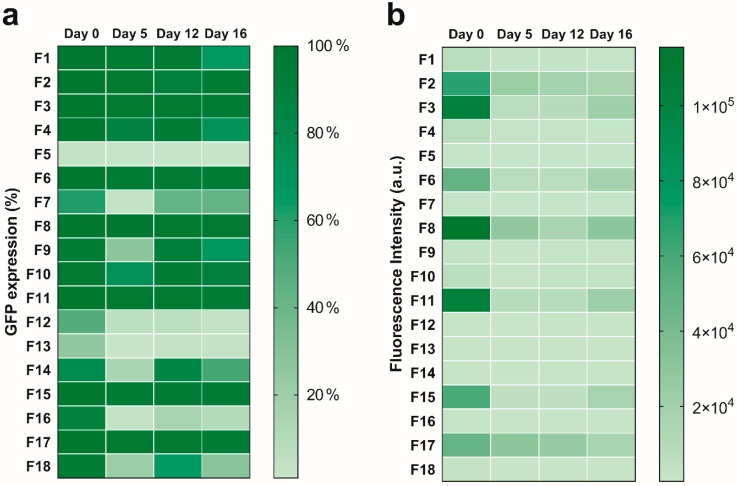
Efficiency of intracellular protein expression in HEK-293 cells over 16 days after LNP preparation. (**a**) percent GFP expression, and (**b**) fluorescence intensity.

The intracellular protein expression of pre- and post-nebulized LNP mRNA formulations was assessed using flow cytometry by measuring percent GFP expression and fluorescence intensity in HEK-293 and NuLi-1 cell lines. On day 0 (i.e., incubation same day as preparation of formulations), we measured the intracellular protein expression of each mRNA-encapsulated formulation in HEK-293 cells to identify formulations that exhibited higher transfection than the reference formulation (DLin-MC3-DMA: DSPC: cholesterol: PEG-DMG = 50: 10: 38.5: 1.5, N/P = 15). It was found that most formulations showed over 50% GFP expression, except F5, F12, and F13. Notably, although most formulations had relatively high percent GFP expression, the intracellular protein expression in terms of fluorescence intensity varied among the formulations. Eight out of 18 formulations (F2, F3, F4, F6, F8, F11, F15 and F17) showed a significantly higher fluorescence intensity compared to the reference formulation, which showed a mean fluorescence intensity of 6708 a.u. in HEK-293 cells on Day 0. The percent GFP expression of these eight formulations were as high as over 95% and showed no significant differences when compared to the reference formulation. Next, the shelf stability of LNPs was tested by quantifying their intracellular protein expression after 0, 5, 12, and 16 days of refrigerated storage. As shown in Figure 2a, eight formulations (F2, F3, F6, F8, F10, F11, F15 and F17) remained stable in terms of percent GFP expression after 16 days of storage at 4 °C. In contrast, the fluorescence intensity of all formulations decreased significantly after 5 days of storage at 4 °C (Figure 2b). Specifically, F2, F3, F6, F8, F11, F15, and F17 showed fluorescence intensities of over 18,000 a.u. which were significantly higher than the reference formulation after 16 days of storage.

**Figure 3:**
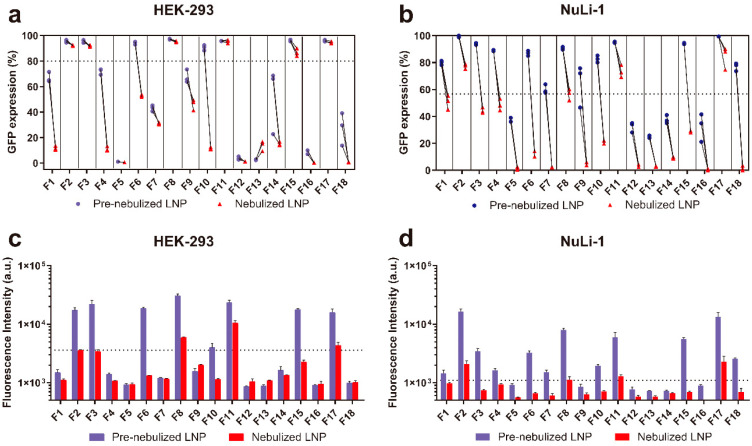
In vitro intracellular protein expression in terms of percent GFP expression and fluorescence intensity of LNP formulations before and after nebulization in HEK-293 (**a**,**c**) and NuLi-1 cells (**b**,**d**). Figure 3a,b represent the percent GFP expression in HEK-293 and NuLi-1 cells, respectively. Figure 3c,d represent the GFP fluorescence intensity in HEK-293 and NuLi-1 cells, respectively.

Upon nebulization, all LNP formulations showed significantly decreased fluorescence intensity compared to pre-nebulized LNP formulations in both HEK-293 cells and NuLi-1 cells. This finding indicates that the aerosolization process negatively affected the mRNA transfection in vitro. We found that F2, F3, F8, F11, and F17 showed no significant changes in terms of percent GFP expression after nebulization compared to pre-nebulized LNPs (Figure 3a,c). Despite a significant decrease in fluorescence intensity observed in all LNP formulations, the aforementioned five formulations retained relatively high fluorescence intensity (over 3000 a.u.) upon nebulization (Figure 3b,d). In NuLi-1 cells, although F2, F8, F11, and F17 showed decreased percent GFP expression and fluorescence intensity upon nebulization, these four formulations still demonstrated relatively high GFP expression (over 50%) and fluorescence intensity (over 1000 a.u.) compared to other formulations. In summary, four formulations (F2, F8, F11 and F17) with relatively high intracellular protein expression after 16 days of storage and nebulization were identified.

### 3.4. Intratracheal Delivery of LNP Formulations to Mice

**Figure 4:**
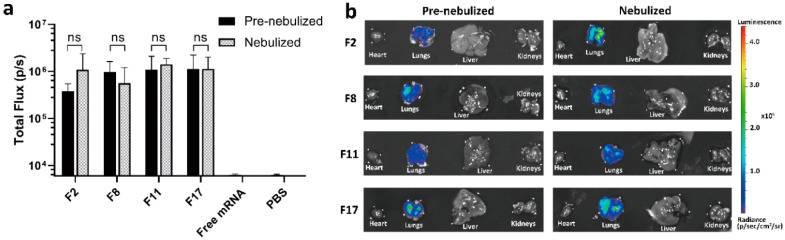
Delivery efficacy of F2, F8, F11, F17 formulations with luciferase mRNA. (**a**) Efficacy of the four lead formulations before and after nebulization in lung as measured in total flux of luminescence 6 h after intratracheal delivery of 1.5 µg of total mRNA. (**b**) Representative images of the luciferase expression in the lungs, heart, liver, and kidneys measured by in vivo imaging system (IVIS) imaging.

Based on intracellular protein expression in vitro, four lead formulations (F2, F8, F11 and F17) were selected for further study in vivo. Specifically, firefly luciferase (Luc) reporter mRNA was loaded into these LNP formulations and nebulized by an Aerogen Solo nebulizer. The collected nebulized dispersions were compared to pre-nebulized controls using intratracheal instillation administration to the lungs of mice to investigate in vivo transfection. Interestingly, there was no statistically significant difference in luminescence intensity between mice dosed with either pre-nebulized or nebulized LNP formulations (Figure 4a), which indicated that the candidate formulations retained their potency after nebulization. After 6 h post-administration, luciferase activity was predominantly detected in the lungs compared to the heart, liver, and kidneys for the four lead formulations, irrespective of the nebulization process (Figure 4b). Free mRNA and PBS did not show luciferase activity (Figure S5).

The Luc mRNA expression in vivo was tested up to 24 h, and the 6 h timepoint demonstrated the highest mRNA expression signal post-administration. Previously, lipid nanoparticles encapsulating Luc mRNA have been evaluated for protein expression and biodistribution in vivo 6 h after intravenous injection [7,11,12,29].

## 4. Discussion

This work highlights a DOE approach to discover LNP-mRNA formulations that can be aerosolized for pulmonary delivery. One-factor-at-a-time design methods have been employed in several studies to investigate the effect of formulation composition on the efficacy of each LNP formulation [34,35]. However, this approach does not account for potential second-order interactions between composition parameters, which makes it less desirable for optimization of LNP formulations. Alternatively, fractional factorial design has been used to maximize the potency of LNP formulations for mRNA delivery [29]. Although this method investigates second-order effects, the fact that not all variables can be included in the design is a major limitation. In order to systematically investigate the effects of variables on the potency of LNP formulations, a mixture design with constraints was employed in this study (Table S1). Using DOE, 18 formulations of various lipid compositions were prepared and characterized in terms of physicochemical properties and intracellular protein expression. The experimental design space for each composition was determined based on previously published reports related to LNP compositions. In order to explore the LNP composition’s effects on LNP nebulization, we decided to broaden the molar ratio range of each LNP composition based on previous studies [21,28,36,37,38]. Four lead formulations that had relatively higher intracellular protein expression than other formulations, before and after nebulization, were identified. These LNP-mRNA formulations (pre- and post-nebulized) were intratracheally delivered to mice for pulmonary delivery and assessed for in vivo expression. We applied different amounts of mRNA for the in vivo studies to reflect the difference in cell surface area in a 96-well plate versus that of epithelial cells in the lungs. Following in vivo studies, extensive statistical analysis of formulations helped identify certain parameters that may impact the stability and intracellular delivery of our nanoparticles.

Here, the measured protein expression reflected the result of a combination of extracellular and intracellular processes, including cellular uptake, trafficking, and endosomal escape. It is of high interest to evaluate these mechanisms separately. In addition, it has been previously demonstrated that different lipids such as helper lipids, cholesterol, and PEGylated-lipids play a role in the lipid particle morphology, optimizing LNP stability and possibly performance [39,40]. Future studies will investigate mechanisms of intracellular uptake and endosomal escape of the tested formulations on mRNA delivery and expression, as well as the effects of different N/P ratios and particle morphology on stability and transfection efficiency. Moreover, future studies will evaluate size and zeta-potential after incubation with bronchoalveolar lavage fluid to assess particle stability in this relevant fluid present in the lungs.

### 4.1. Composition of LNP Formulations Influenced Their Physicochemical Properties (Size, Zeta Potential, and Encapsulation Efficiency) before and after Nebulization

The particle sizes increased after nebulization for all the LNP formulations studied. The underlying mechanism may be due to shear forces, interfacial and thermal stresses during the nebulization process [41]. It was found that pre-nebulized dispersions had a particle size that was dependent on the molar ratio of PEG-lipid used. In these pre-nebulized formulations, it appeared that the type of PEG-lipid used did not influence particle size in a significant way. In contrast, the nebulized dispersions were significantly influenced by the type of PEG-lipid used in the formulation. These observations are discussed below.

**Figure 5:**
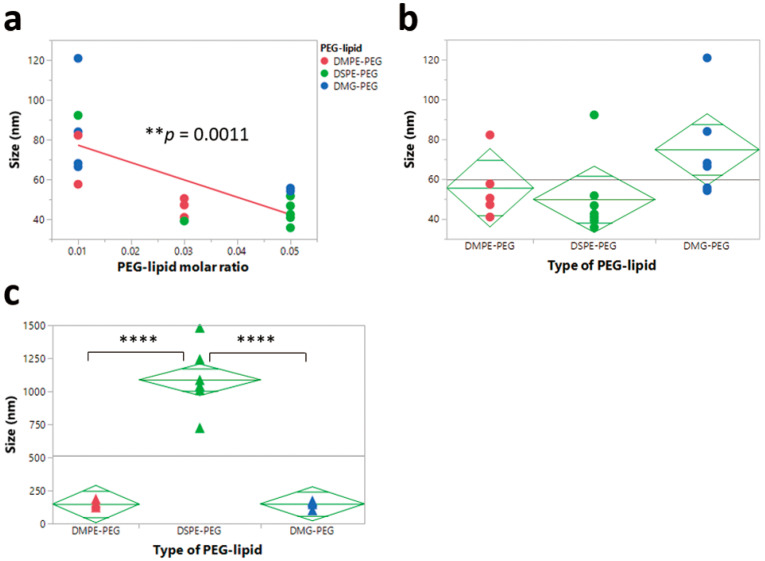
Correlation between particle size and poly-(ethylene) glycol (PEG)-lipid. (**a**) Effects of PEG-lipid molar ratio on particle size before nebulization. (**b**) Effects of type of PEG-lipid on particle size before nebulization (**c**) Effects of type of PEG-lipid on particle size after nebulization. (** *p* < 0.01, **** *p* < 0.0001).

To explore the correlation between LNP size and each LNP component, the size of the LNPs before and after nebulization was plotted against each component, and the orthogonal trend was analyzed. A statistically significant (*p* < 0.05) trend of decreasing size was observed with increasing molar PEG-lipid composition for pre-nebulized LNP formulations, independent of the other formulation parameters (Figure 5a). The size was not significantly correlated to other components of the formulation in terms of molar amounts. Similar findings have been reported where PEGylated liposomes showed a significant decrease in size compared to conventional liposomes, and that increasing the overall amount of DSPE-PEG led to a decrease in liposome size [42,43]. A potential explanation for this finding could be due to the fact that lateral repulsion of the surface of lipid bilayers increases by extensive hydration around the head group with an increasing concentration of PEG-lipid. To reduce the high lateral repulsion, particle sizes must decrease, which subsequently increases the curvature of the grafting surface [43]. In post-nebulization formulations, formulations with DSPE-PEG showed a larger size compared to formulations with either DMPE-PEG or DMG-PEG, which indicated that the type of PEG-lipid significantly affected the size of LNPs after nebulization (Figure 5c). These results indicated that formulations made with DSPE-PEG had a poor ability to maintain their size after the aerosolization process. The zeta potential of the formulations, both before and after nebulization, was also primarily driven by the type of PEG-lipid selected, where formulations with DSPE-PEG showed a higher zeta potential, irrespective of aerosolization (Figure S3a,b).

**Figure 6:**
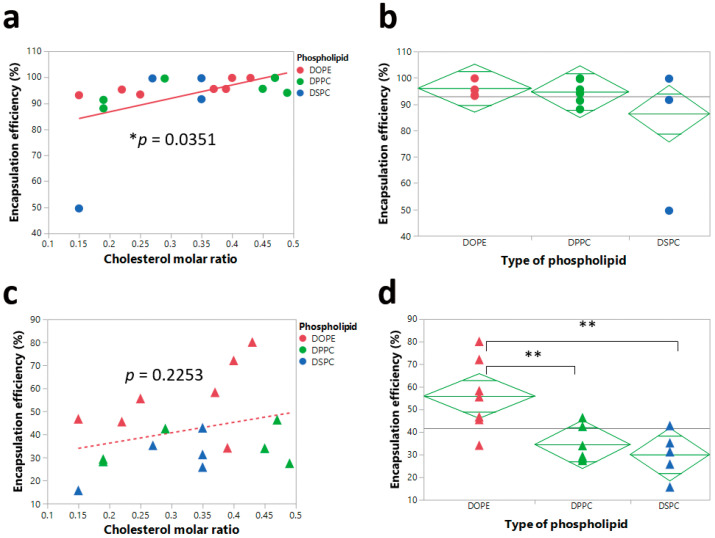
Correlation of encapsulation efficiency and cholesterol molar ratio and type of phospholipid. (**a**) Significant correlation (* *p* < 0.05) between encapsulation efficiency and cholesterol molar ratio before nebulization. (**b**) No significant effects (*p* > 0.05) of type of phospholipid on encapsulation efficiency before nebulization. (**c**) No significant correlation between encapsulation efficiency and cholesterol molar ratio after nebulization. (**d**) Significant effects of type of phospholipid on encapsulation efficiency after nebulization. ** *p* < 0.01.

With respect to encapsulation efficiencies, almost all the formulations achieved high encapsulation efficiency. Increasing the cholesterol molar ratio resulted in a statistically significant increase in the encapsulation efficiency for the pre-nebulized LNPs (Figure 6a). This suggests that the structural cholesterol may play an important role in the encapsulation efficiency of LNP formulations before aerosolization, while the type of phospholipid used did not demonstrate significant effects before nebulization (Figure 6b). It has been previously reported that lipid-like nanoparticles with higher molar ratios of cholesterol possessed higher encapsulation efficiencies of mRNA [44]. However, after nebulization, the type of phospholipid, instead of the molar amount of cholesterol, became the factor that significantly influenced the encapsulation efficiency (Figure 6c,d). LNP formulations with DOPE showed a significantly higher encapsulation efficiency compared to LNP formulations with either DSPC or DPPC (Figure 6d). This finding indicates that the inclusion of DOPE could significantly enhance the ability of LNPs to retain mRNA during the aerosolization process.

### 4.2. PEG-Lipid Molar Ratio Negatively Influenced the Intracellular Protein Expression of LNPs before and after Nebulization

Formulations of the mRNA loaded LNPs must balance several performance measures, such as transfection efficiencies and nanoparticle stability. In the formulations developed in this study, PEG-lipids were used to impart physical stability on the nanoparticle dispersion. However, it has been shown that PEGylation can significantly influence transfection efficiencies [45,46,47]. Here, the PEG-lipid molar ratio significantly and negatively affected the intracellular protein expression of LNPs both before and after nebulization.

**Figure 7:**
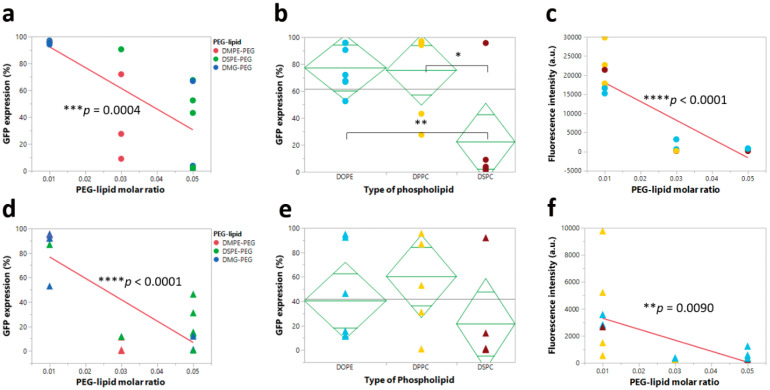
Correlation analysis between intracellular protein expression (percent GFP expression and fluorescence intensity) and PEG-lipid molar ratio or type of phospholipid. (**a**) Significant effect of PEG-lipid molar ratio on percent GFP expression before nebulization. (**b**) Significant effect of type of phospholipid on percent GFP expression before nebulization. (**c**) Significant effect of PEG-lipid molar ratio on fluorescence intensity before nebulization. (**d**) Significant effect of PEG-lipid molar ratio on percent GFP expression after nebulization. (**e**) No significant effect of type of phospholipid on percent GFP expression after nebulization. (**f**) Significant effect of PEG-lipid molar ratio on fluorescence intensity after nebulization (* *p* < 0.05, ** *p* < 0.01, *** *p* < 0.001, and **** *p* < 0.0001).

Specifically, increasing the PEG-lipid molar ratio negatively affected the intracellular protein expression of pre-nebulized LNPs in HEK-293 cells (Figure 7a,c) and NuLi-1 cells (Figure S4). A statistically significant trend of decreasing percent GFP expression and fluorescence intensity was observed with an increasing molar fraction of PEG-lipid, independent of the other formulation parameters; this finding was consistent with previous reports [45,47]. In addition, the type of phospholipid significantly influenced percent GFP expression. We observed that LNP formulations with DSPC showed significantly lower percent GFP expression compared to LNP formulations with either DOPE or DPPC (Figure 7b), an observation consistent with previous reports [29]. Upon nebulization, increasing the molar ratio of PEG-lipid resulted in the same observed trend in HEK-293 (Figure 7d,f) and NuLi-1 cells (Figure S4), but there were no significant effects of the type of phospholipid on percent GFP expression (Figure 7e).

### 4.3. Correlation between Physicochemical Properties and Intracellular Protein Expression before and after Nebulization

**Figure 8:**
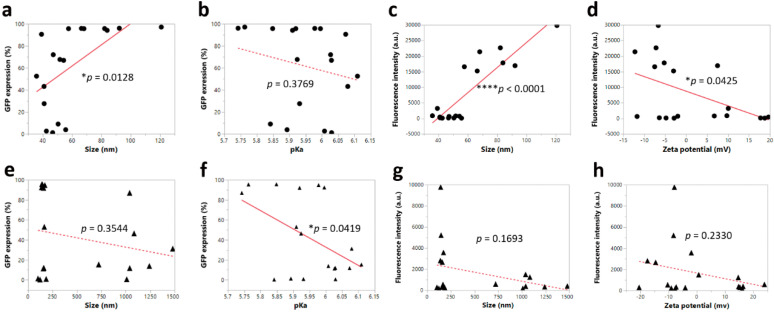
Orthogonal trends of intracellular protein expression in terms of percent GFP expression and fluorescence intensity, whereby dotted line represented non-significance and solid line represented significance. (**a**–**d**): Correlation between intracellular protein expression and formulation properties before nebulization. (**e**–**h**): Correlation between intracellular protein expression and formulation properties after nebulization (* *p* < 0.05, and **** *p* < 0.0001).

In order to explore the correlation between physicochemical properties and the potency of LNP formulations, we plotted size, zeta potential, encapsulation efficiency, and pKa against intracellular protein expression and fluorescence intensity in HEK-293 cells. It was found that LNP formulations with a larger particle size showed a higher uptake, as evidenced by a higher percent GFP expression and fluorescence intensity before nebulization (Figure 8a,c). Furthermore, pre-nebulized formulations with a higher zeta potential showed a lower fluorescence intensity (Figure 8d). After nebulization, the pKa appeared to be the significant parameter influencing percent GFP expression, whereby a lower pKa led to a higher percent GFP expression (Figure 8f), while other parameters showed no significant effects on the intracellular protein expression. Interestingly, it has been reported that LNPs with a higher pKa value demonstrated a higher in vivo potency when the pKa ranged from 4.17 to 6.44. The possible underlying reason is that their optimal LNP with a pKa value of 6.4 was able to have a minimum positive charge in the blood at pH 7.4 and a maximum positive charge in acidified endosomes at pH 5.5 [28]. However, in our studies, the significant correlation between pKa and percent GFP expression was only observed in nebulized LNPs while the unnebulized LNPs showed no significant correlation. This discrepancy may be due to the disruption of LNPs during the nebulization process, which could have induced a change in surface charge.

For effective pulmonary drug delivery, LNP-mRNA formulations must be stably aerosolized. Thus, we investigated the transfection efficiency of the four lead formulations (F2, F8, F11, and F17) with luciferase mRNA in vivo after intratracheal delivery (1.5 µg dose per mouse) before and after nebulization. Both pre-nebulized and nebulized formulations expressed luciferase in the mouse lungs at 6 h post-administration, with no significant differences observed between the two groups (Figure 4a). On the other hand, naked free mRNA failed to transfect and express luciferase in the mouse lungs, which is in agreement with previous studies that demonstrated poor transfection of naked mRNA in the lungs after intratracheal administration [11]. Compared with the in vitro studies, we observed similar trends within these four lead formulations in HEK-293 cells, where the % GFP expression changes before and after nebulization were negligible (Figure 3a), while in NuLi-1 cells although the nebulization process decreased the % GFP expression, these four lead formulations still demonstrated the highest transfection efficiencies after nebulization (Figure 3b). Most importantly, the nebulized LNP formulations were able to mediate effective luciferase mRNA transfection in the mouse lungs, without any evidence of transfection in the heart, liver, and kidneys. These findings have clinical implications due to the LNPs desirable stability during aerosolization and gene transfer potency for therapeutic pulmonary delivery. This is the first study demonstrating retained potency between both unnebulized and nebulized LNP-mRNA formulations in vivo after intratracheal administration.

## 5. Conclusions

In this study, using the DOE approach for formulation discovery helped to identify certain parameters that impacted the stability and intracellular delivery of nanoparticles. The in vitro performance of LNP formulations for aerosol gene delivery was significantly influenced by lipid composition. DSPE-PEG was a negative factor for the stability of LNP nanoparticles as a significantly higher aggregate level appeared after nebulization compared to formulations with DMG-PEG and DMPE-PEG. It was also found that the PEG-lipid molar ratio and DSPC phospholipid significantly and negatively affected the intracellular protein expression of LNPs. Moreover, four lead formulations that had relatively higher intracellular protein expression than other formulations before and after nebulization were identified and subsequently tested in vivo. The pre- and post-nebulized formulations, when intratracheally delivered to mice, delivered mRNA to the lungs for in vivo expression. From this approach, LNP formulations can be more rapidly and easily identified that possess the optimal properties to facilitate effective aerosolized delivery of mRNA. While this work focused on the delivery of mRNA towards the treatment of pulmonary diseases, the DOE strategy could be broadly applied to discover LNP compositions and their properties that promote enhanced delivery of nucleic acid therapeutics for different indications in gene therapy.

## Supplementary Materials

The following are available online at https://www.mdpi.com/1999-4923/12/11/1042/s1, Table S1: Limits of experimental design space, Table S2: LNP compositions for 18 formulations, Table S3: PDI of pre-nebulized LNP at Day 1 and Day 14, and nebulized LNP, Figure S1: Intracellular protein expression of LNP formulations at different N/P ratios in HEK-293 cells measured by percent GFP expression (left axis) and fluorescence intensity (right axis), Figure S2: Characterization of LNP formulations. Figure S3: Significant effects of type of PEG-lipid on zeta potential before (a) and after (b) nebulization, Figure S4: Correlation analysis between intracellular protein expression (percent GFP expression and fluorescence intensity) and PEG-lipid molar ratio or type of phospholipid in NuLi-1 cells. Figure S5: Representative images of the luciferase expression in lungs, heart, liver, and kidneys in negative control groups measured by IVIS imaging.
