# Supplementary material for: Aerosolizable Lipid Nanoparticles for Pulmonary Delivery of mRNA through Design of Experiments
Source: Pharmaceutics. 2020 Oct 30;12(11):1042. doi: 10.3390/pharmaceutics12111042 (PMC7692784; doi:10.3390/pharmaceutics12111042)
Supplement: Supplementary file 1 [file pharmaceutics-12-01042-s001.pdf]

pharmaceutics

MDPI

Supplementary Material

Aerosolizable Lipid Nanoparticles for Pulmonary Delivery of mRNA through Design of Experiments

Hairui Zhang†, Jasmim Leal†, Melissa R. Soto, Hugh D.C. Smyth, Debadyuti Ghosh*

Division of Molecular Pharmaceutics and Drug Delivery, College of Pharmacy, The University of Texas at Austin, Austin, Texas 78712, United States

* Correspondence: dghosh@austin.utexas.edu; Tel.: +01-512-232-7155

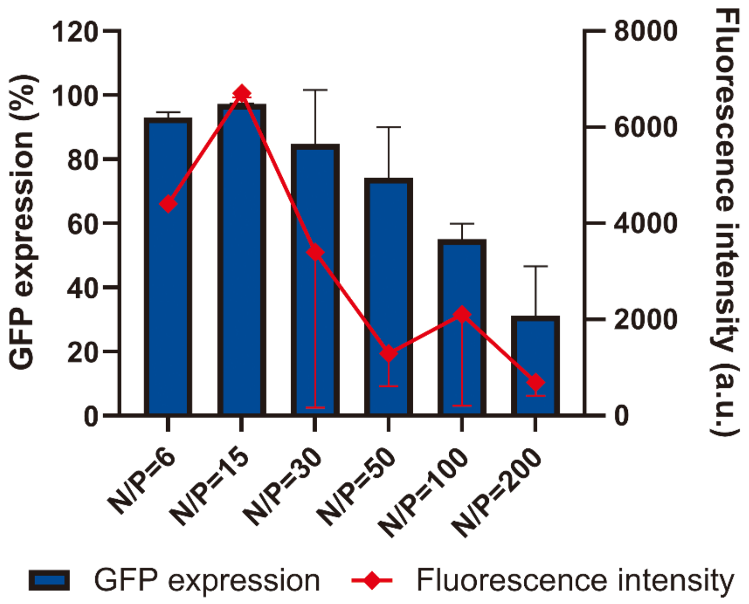

Figure S1: Intracellular uptake of LNP formulations at different N/P ratios in HEK-293 cells measured by percent GFP expression (left axis) and fluorescence intensity (right axis).

Table S1: Limits of experimental design space

Component	Lower limit (molar ratio)	Upper limit (molar ratio)
Ionizable lipid	0.4	0.6
Phospholipid	0.1	0.2
PEG-lipid	0.01	0.05
Cholesterol	0.15	0.49

Table S2: LNP compositions for 18 formulations.

Formulation #	Phospholipid	PEG-lipid	Molar composition			
			Dlin-MC3-DMA	Phospholipid	PEG-lipid	Cholesterol
**1**	DOPE	DMG-PEG	0.6	0.2	0.05	0.15
**2**	DOPE	DMPE-PEG	0.4	0.2	0.01	0.39
**3**	DSPC	DMG-PEG	0.5	0.14	0.01	0.35
**4**	DOPE	DMPE-PEG	0.6	0.15	0.03	0.22
**5**	DSPC	DSPE-PEG	0.4	0.2	0.05	0.35
**6**	DPPC	DMG-PEG	0.4	0.1	0.01	0.49
**7**	DPPC	DSPE-PEG	0.4	0.1	0.05	0.45
**8**	DPPC	DMG-PEG	0.6	0.2	0.01	0.19
**9**	DOPE	DSPE-PEG	0.6	0.1	0.05	0.25
**10**	DOPE	DSPE-PEG	0.4	0.2	0.03	0.37
**11**	DPPC	DMPE-PEG	0.6	0.2	0.01	0.19
**12**	DSPC	DMG-PEG	0.6	0.2	0.05	0.15
**13**	DSPC	DSPE-PEG	0.5	0.1	0.05	0.35
**14**	DOPE	DSPE-PEG	0.4	0.15	0.05	0.4
**15**	DPPC	DSPE-PEG	0.6	0.1	0.01	0.29
**16**	DSPC	DMPE-PEG	0.5	0.2	0.03	0.27
**17**	DOPE	DMG-PEG	0.4	0.16	0.01	0.43
**18**	DPPC	DMPE-PEG	0.4	0.1	0.03	0.47

Table S3: PDI of pre-nebulized LNP at Day 1 and Day 14, and nebulized LNP.

Formulation #	PDI		
	Pre-nebulized LNP - Day 1	Pre-nebulized LNP - Day 14	Nebulized LNP
**1**	0.261 ± 0.005	0.278 ± 0.022	0.245 ± 0.051
**2**	0.093 ± 0.009	0.128 ± 0.009	0.232 ± 0.024
**3**	0.080 ± 0.004	0.101 ± 0.004	0.268 ± 0.005
**4**	0.161 ± 0.006	0.172 ± 0.014	0.293 ± 0.008
**5**	0.205 ± 0.006	0.365 ± 0.151	0.591 ± 0.076
**6**	0.035 ± 0.029	0.042 ± 0.015	0.366 ± 0.058
**7**	0.241 ± 0.010	0.486 ± 0.029	0.425 ± 0.017
**8**	0.041 ± 0.002	0.045 ± 0.026	0.128 ± 0.009
**9**	0.199 ± 0.013	0.208 ± 0.033	0.402 ± 0.050
**10**	0.155 ± 0.017	0.197 ± 0.008	0.507 ± 0.062
**11**	0.058 ± 0.012	0.082 ± 0.010	0.235 ± 0.005
**12**	0.111 ± 0.015	0.188 ± 0.008	0.220 ± 0.008
**13**	0.149 ± 0.005	0.429 ± 0.025	0.668 ± 0.131
**14**	0.240 ± 0.006	0.343 ± 0.010	0.877 ± 0.114
**15**	0.057 ± 0.017	0.142 ± 0.005	0.575 ± 0.090
**16**	0.115 ± 0.010	0.157 ± 0.003	0.231 ± 0.013
**17**	0.121 ± 0.005	0.135 ± 0.009	0.213 ± 0.005
**18**	0.214 ± 0.007	0.366 ± 0.008	0.310 ± 0.025

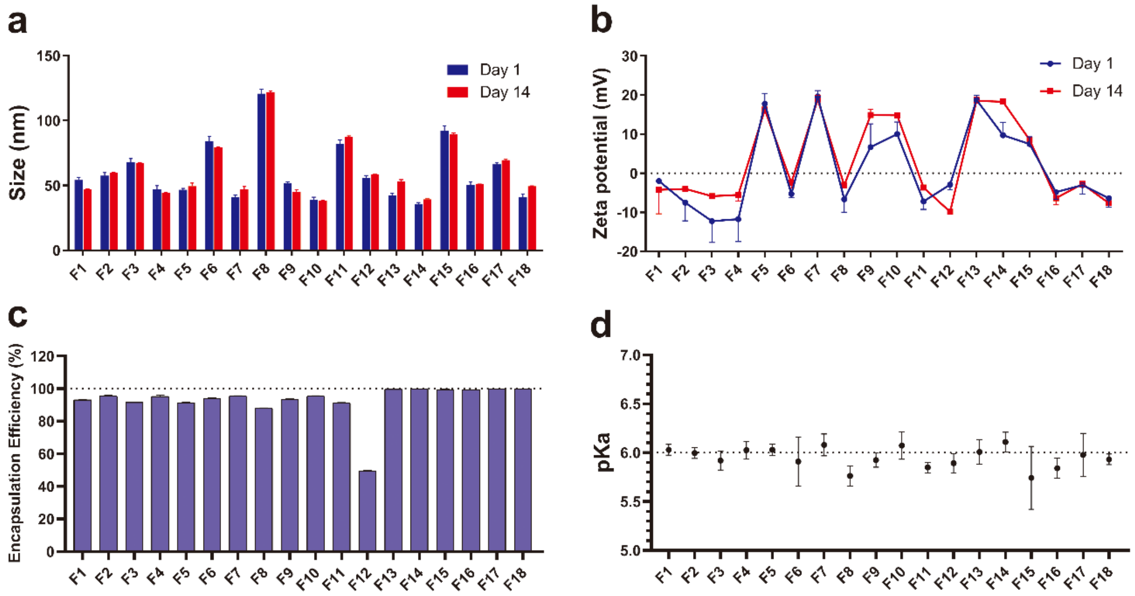

Figure S2: Characterization of LNP formulations. (a) size, (b) zeta-potential, (c) encapsulation efficiency, and (d) pKa. Stability of the lipid nanoparticles was evaluated by measuring size and zeta-potential at day 1 and after 14 days from preparation and storage at 4 ◎C. (mean ± SD, n=3).

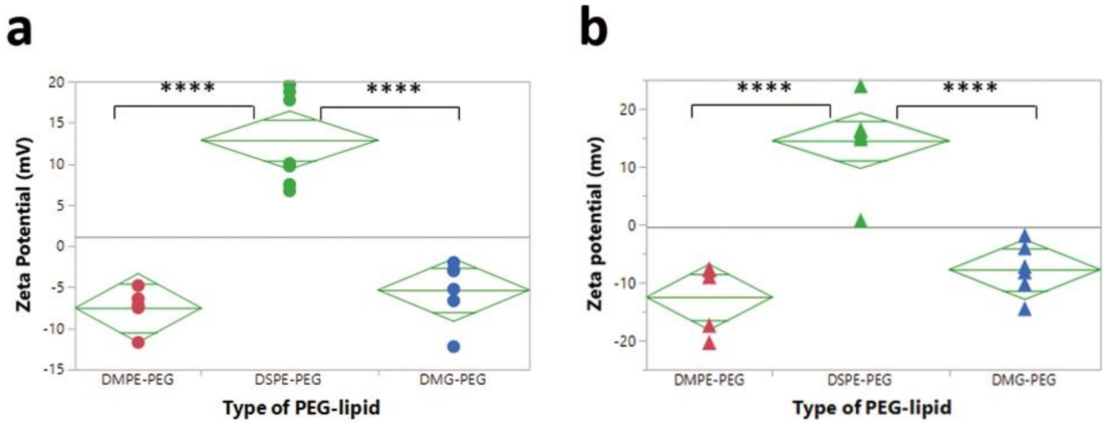

Figure S3: Significant effects of type of PEG-lipid on zeta potential before (a) and after (b) nebulization.

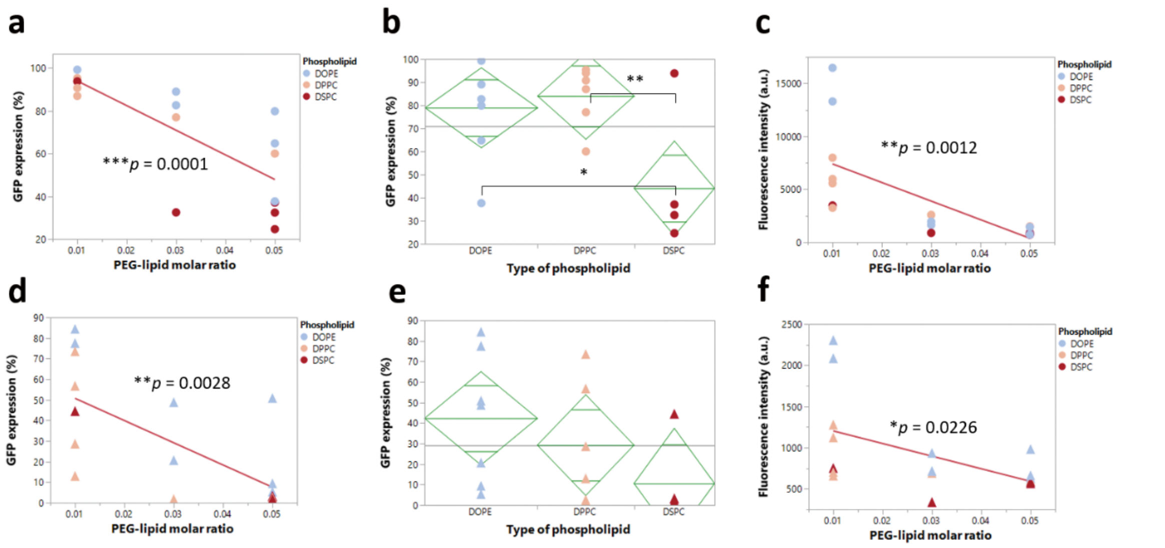

Figure S4: Correlation analysis between intracellular uptake (percent GFP expression and fluorescence intensity) and PEG-lipid molar ratio or type of phospholipid in NuLi-1 cells. Significant effect of PEG-lipid molar ratio (a) and type of phospholipid (b) on the percent GFP expression before nebulization. (c) Significant effect of PEG-lipid molar ratio on the fluorescence intensity before nebulization. Significant effect of PEG-lipid molar ratio (d) and no significant effect of type of phospholipid (e) on the percent GFP expression after nebulization. (f) Significant effect of PEG-lipid molar ratio on fluorescence intensity after nebulization.

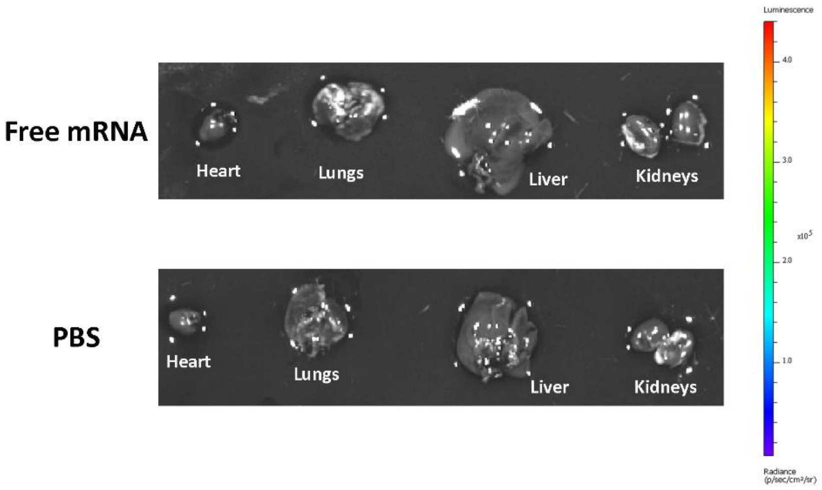

Figure S5: Representative images of the luciferase expression in lungs, heart, liver, and kidneys in negative control groups measured by IVIS imaging.
